# The Role of Gut Microbiota and Its Produced Metabolites in Obesity, Dyslipidemia, Adipocyte Dysfunction, and Its Interventions

**DOI:** 10.3390/metabo11080531

**Published:** 2021-08-10

**Authors:** Max S. Z. Zwartjes, Victor E. A. Gerdes, Max Nieuwdorp

**Affiliations:** 1 Department of Experimental Vascular Medicine, Amsterdam University Medical Center, 1105 AZ Amsterdam, The Netherlands; v.e.gerdes@amsterdamumc.nl (V.E.A.G.); m.nieuwdorp@amsterdamumc.nl (M.N.); 2 Department of Internal Medicine, Spaarne Gasthuis, Spaarnepoort 1, 2134 TM Hoofddorp, The Netherlands; 3 Department of Internal and Vascular Medicine, Amsterdam University Medical Center, 1105 AZ Amsterdam, The Netherlands; 4 Department of Internal Medicine, Diabetes Center, Amsterdam University Medical Center, 1105 AZ Amsterdam, The Netherlands

**Keywords:** dyslipidemia, lipid metabolism, obesity, gut microbiota, metabolites, lipoprotein lipase, reverse cholesterol transport, bile acid receptors, adipocyte dysfunction, adipose tissue inflammation, lipid-lowering agents

## Abstract

Obesity is becoming an increasing problem worldwide and is often, but not invariably, associated with dyslipidemia. The gut microbiota is increasingly linked to cardiovascular disease, nonalcoholic fatty liver disease, and type 2 diabetes mellitus. However, relatively little focus has been attributed to the role of gut-microbiota-derived metabolites in the development of dyslipidemia and alterations in lipid metabolism. In this review, we discuss current data involved in these processes and point out the therapeutic potentials. We cover the ability of gut microbiota metabolites to alter lipoprotein lipase action, VLDL secretion, and plasma triglyceride levels, and its effects on reverse cholesterol transport, adipocyte dysfunction, and adipose tissue inflammation. Finally, the current intervention strategies for treatment of obesity and dyslipidemia is addressed with emphasis on the role of gut microbiota metabolites and its ability to predict treatment efficacies.

## 1. Introduction

Obesity has approximately tripled from 1975 to 2016, affecting 650 million adults worldwide, and in 2016, 39% of the world population was overweight [1]. Although obesity prevalence remains highest in high-income countries, obesity has increased in each and every World Bank income group and is becoming a rising problem in developing countries [2,3]. Obesity increases the risk of insulin resistance, glucose intolerance and type 2 diabetes mellitus (T2DM), nonalcoholic fatty liver disease (NAFLD), several types of cancer including breast, endometrial, and colorectal cancer, obstructive sleep apnea (OSA), and as discussed in this review, obesity is frequently accompanied by dyslipidemia with subsequent atherosclerotic cardiovascular disease (ASCVD) [4].

One of the most important triggers for atheroma formation within arterial walls and the resulting ASCVD is dyslipidemia, characterized by abnormal elevations and deposition of mainly Apo-B-containing lipoproteins including low-density lipoprotein cholesterol (LDL-C) [5,6]. The main reason we treat dyslipidemias is to prevent ASCVD, including coronary artery disease and stroke [7]. Since cardiovascular diseases continue to be the most common cause of mortality in low- to high-income countries [8] and alterations in the lipid profile are seen as one of its primary causes, the search for further pathogenetic mechanisms of dyslipidemia is highly relevant. Obesity is often associated with dyslipidemia; however, many patients with severe obesity do not have characteristics of dyslipidemia and although the designation of patients as ‘metabolically healthy’ is a topic of ongoing discussion, many patients with obesity might have underlying protective mechanisms against cardiometabolic disease, which remain to be discovered [9].

Recent research has increasingly focused on the role of gut microbiota (GM) and its metabolites in the pathogenesis of cardiometabolic disease [10,11], including T2DM, ASCVD, and NAFLD, with important microbially produced metabolites including the microbial formed trimethylamine (TMA) and its oxidized form TMAO, lipopolysaccharide (LPS), secondary bile acids, and the more recently discovered association of phenylacetylglutamine (PAG) and its role in thrombosis. However, relatively little interest has been paid to the possible role of gut microbiota and its produced metabolites in lipid metabolism in obesity and the development of dyslipidemia.

## 2. Obesity and Dyslipidemia

A surplus of caloric intake combined with a sedentary lifestyle and genetic predisposition causes the expansion of adipose tissue, termed obesity. Excess energy is efficiently stored as triglycerides within our adipocyte depots, and if required, energy can be released by lipolysis of triglycerides into free fatty acids (FFAs). Before discussing definitions of dyslipidemia and its pathogenetic mechanisms in obesity, we describe lipid metabolism in healthy persons.

### 2.1. Normal Lipid Metabolism

**Figure 1:**
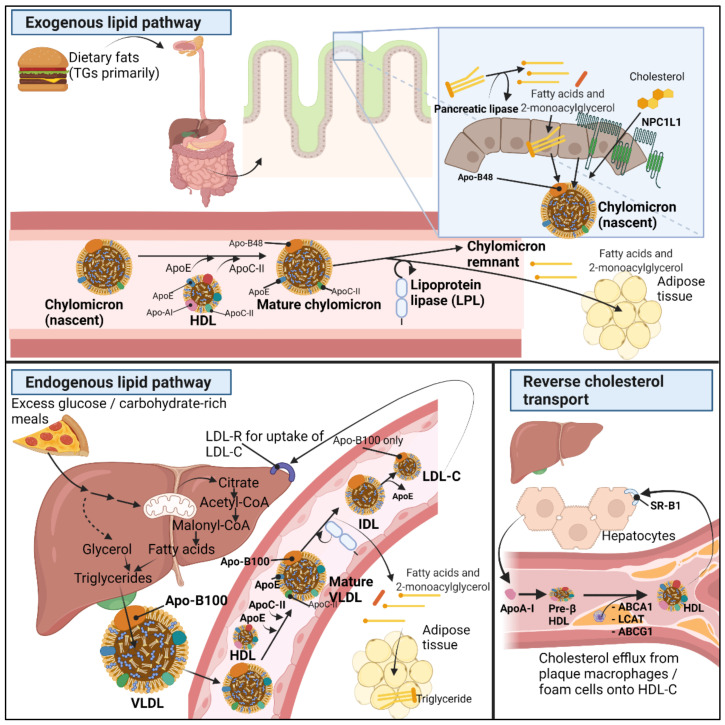
Normal lipid metabolism. Lipid metabolism is divided into exogenous (**top**) and endogenous (**bottom left**) lipid metabolism. Reverse cholesterol transport (**bottom right**) is crucial for the return of cholesterol from peripheral tissues to the liver. Further description in main text above Section 2.2. Metabolic health and dyslipidemia in patients with obesity.

The circulating lipoproteins in our plasma are aggregate molecules composed of a lipid core with different concentrations of triglycerides and cholesteryl esters packed inside a phospholipid monolayer with scattered free cholesterol and surface apolipoproteins [12]. The contents inside the lipoproteins can be exogenous such as for chylomicrons synthesized by enterocytes or endogenous such as for very low-density lipoprotein (VLDL) synthesized by hepatocytes. Intermediate (IDL) and low-density lipoproteins (LDL) cholesterol are derived from further enzymatic steps [12]. Figure 1 summarizes the exogenous, endogenous, and reverse cholesterol transport mechanisms.

Exogenous lipids are the fats absorbed from our diet. The ingested lipids, primarily triglycerides (TGs), are turned into fatty acids (FAs) and 2-monoacylglycerol (2-MAG) by pancreatic lipase at the intestinal lumen [13]. Next, long-chain fatty acids, MAGs, cholesterol, and other lipid digestion-derived molecules are re-esterified inside enterocytes, forming TGs, phospholipids, and cholesteryl esters. In order to solubilize TGs and the cholesteryl esters, enterocytes pack lipids into apolipoprotein B48-positive (Apo-B48) chylomicrons released into lymph and circulation [14]. Dietary cholesterol uptake occurs with the help of Niemann–Pick C1-like 1 protein (NPC1L1) before cholesteryl ester formation and transport into chylomicrons [15]. Next, after chylomicron maturation by action of HDL (see below), mature chylomicrons are formed from which lipoprotein lipase can draw out free fatty acids, leaving chylomicron remnants through which ApoE binding to hepatocyte expressed receptors enters and is broken down within hepatocytes [16]. Notably, some products of luminal lipid digestion are water soluble and hence transported readily across the enterocyte via several membrane transporters to enter the portal blood; these products include glycerol, short-, and medium-chain fatty acids (SCFA, MCFA) [17].

The other class of lipids are endogenous lipids, formed under the high influence of insulin [18]. Endogenous TGs and other lipids are released as very low density lipoprotein (VLDL) by hepatocytes; after intake of a carbohydrate-rich meal, after further caloric intake when hepatic glycogen stores have already been repleted, excess glucose is converted into pyruvate [19]. Pyruvate inside the mitochondria converts into Acetyl-CoA, to enter the citric acid cycle (CAC) and fuse with oxaloacetate forming citrate, and citrate is pushed back into the cytoplasm, as during the further caloric intake, ATP and NADH molecules have already been produced in high numbers, causing citrate to exit the mitochondria and enter the cytoplasm. Cytosolic citrate once more forms Acetyl-CoA, and by insulin-activated enzymes, acetyl-CoA converts into malonyl-CoA, which eventually turns into fatty acids [18,20]. Cytoplasmic glycerol produced from parallel glycolysis binds with fatty acid, and by several steps, forms TGs. Cholesteryl ester is formed in other steps: in case of decreasing hepatic cholesterol, hepatic sterol regulatory element-binding protein (SREBP2) induces LDL-receptors to the surface in order to bind apoB-100 of lipoproteins such as LDL-C; in addition, SREBP2 induces HMG-CoA reductase for de novo cholesterol synthesis [21]. Altogether, this is de novo lipogenesis, generating endogenous lipids (TG) by the de novo synthesis of fatty acid during the excessive-energy nutrition status. The liver releases triglycerides inside VLDLs, which have apolipoprotein B100 (apoB-100) on its surface [22].

In order for fatty acids to reach the peripheral tissues, lipoprotein lipase (LPL) present on the endothelial surface act on VLDL and chylomicrons to release fatty acids [23]. However, LPL cannot act on these immature chylomicron- or VLDL-molecules; therefore, with the help of high-density lipoprotein (HDL), VLDL and chylomicrons are ‘matured’ as HDL provides the apolipoproteins C-II (ApoC-II) and E (ApoE) [24]. Next, by connecting to ApoC-II, LPL is able to act on the VLDL and chylomicrons to cause the release of its fatty acid contents from its TGs [25]. However, only during high fed states the body wants to store the energy from these fatty acids. Therefore, adipocytes, only under the guidance of insulin-activated genes in adipocytes, eventually cause activation of lipoprotein lipase (LPL) on the surface of the endothelial cells of the adipocyte microcirculation [26]. In such a way, only during high-fed states, LPL takes up the fatty acids, storing TGs by enzymes which esterify fatty acids with glycerol inside adipocytes.

After transfer of TGs from VLDL into the tissues whose microcirculation express LPL and after ApoC-II is returned to HDL, the remnant molecule with often 70–80% of its triglycerides lost but most cholesteryl esters retained is called intermediate-density lipoprotein (IDL) [12]. IDL can either be further relieved of its triglycerides via hepatic lipase forming low-density lipoprotein (LDL-C), or IDL can, by hepatocyte binding with LDL-R to IDL-surface ApoE, be broken down inside hepatocytes [12]. LDL-C has lower levels of Apo-E expressed but still has surface apoB-100 expressed. Parallel to the removal of TGs from VLDL by LPL and/or formation of IDL or LDL, cholesteryl ester transferase protein (CETP) can also act on VLDL and exchange cholesteryl esters, retinyl ester, phospholipids, and triglycerides between VLDL, HDL, and LDL [12,27,28].

The third arm of lipid metabolism is the reverse cholesterol transport (RCT), mediated in part by apolipoprotein AI (Apo-AI)-positive HDL particles which pick up cholesterol from the periphery including from macrophages and foam cells in the blood vessel walls. HDL-C brings cholesterol to the liver for eventual excretion in bile and feces [29,30]. HDL synthesis starts by liver and enterocyte production of Apo-AI, initially devoid of lipids (pre-β-HDL). Next, ATP-binding cassette transporter A1 (ABCA1) ensures the efflux of phosphatidylcholine and cholesterol into the pre-β-HDL to form the discoid form of HDL, a process named HDL maturation [31]. Lecithin cholesterol acyltransferase (LCAT), with the help of cofactor Apo-AI, gives esterification of cholesterol into hydrophobic cholesteryl esters, allowing sequestration of cholesterol from peripheral tissues into the lipid core of HDL. This changes the HDL shape from discoid to its mature spherical form [29,32]. HDL continues to circulate and pick up cholesterol from several peripheral tissues by action of LCAT, ATP-binding cassette transporter G1 (ABCG1), ABCA1, and eventually, HDL returns cholesterol to the liver by action of scavenger receptor class B type 1 (SR-B1) [33].

Although obesity is often complicated by dyslipidemia, there is no absolute association between obesity and dyslipidemia [34], and interestingly, certain patients of the category overweight (BMI 25–29.9 kg/m^2^) have more substantial alterations in the lipid profile than patients with class III obesity (BMI above 40 kg/m^2^) [35]. Some authors state this difference in a proposed disease model of a metabolic ‘healthy obese’ versus metabolically unhealthy normal weight individuals [9,36,37], sometimes called the ‘obesity paradox’ or variations of this terminology [38,39]. In the mentioned studies emphasizing the ‘healthy obese’, the thought is that the risk of developing cardiometabolic disease appears to come from dyslipidemia as a separate entity or from other comorbidities such as insulin resistance and not due to obesity alone. A study by Wildman et al. of the 5440 participants in the National Health and Nutrition Examination Survey (NHANES) from 1999–2003 found that approximately 30% of the obese population and more than 50% of overweight individuals are ‘metabolically healthy’ [40]. The definition of ‘metabolically healthy’ in the mentioned study was one or less of the cardiometabolic risk factor including insulin resistance and diabetes, elevated blood pressure, systemic inflammation, and dyslipidemia [40]. Features of the metabolically healthy obese include conserved insulin sensitivity, low fat mass in visceral adipose tissue, and only minor hepatic fat contents [41]. On a cellular level, according to a review by Müller et al., in obese healthy individuals, the adipose tissue itself is of normal size and without significant hypertrophy, and there is absence of significant inflammatory infiltration by for example macrophages in the adipose tissue [41]. The question whether obesity can be termed ‘healthy’ is however disputed by several studies, and large systematic reviews which have shown obesity as an independent risk factor for ASCVD [42] with higher all-cause mortality of obese individuals without metabolic anomalies as compared to those of normal weight and no metabolic anomalies [43]. In addition, another study by Caleyachetty et al. showed that patients with obesity patients characterized as ‘metabolically healthy’ have a higher risk of cardiovascular disease (coronary heart disease, cerebrovascular disease, and heart failure) as compared to individuals with normal weight and normal metabolic parameters [44]. Furthermore, the state of metabolically healthy obese is thought to be unstable, mainly a feature of younger individuals, and possibly being only an intermediary stage before transition to a state of pathologic (‘unhealthy’) obesity [45].

The dyslipidemia frequently observed in patients with obesity is characterized by elevations in both fasting and postprandial triglycerides, elevated free fatty acids, reduced high-density lipoprotein cholesterol (HDL-C), high-normal to moderately increased low-density lipoprotein cholesterol (LDL-C), and elevations in small-dense LDL [46]. Obesity is also associated with chronic low-grade inflammation, especially by elevations in C-reactive protein (CRP) and other proinflammatory cytokines in obese individuals as compared to lean individuals [47].

### 2.2. Basic Pathogenetic Mechanisms of Dyslipidemia in the Obese Population

Finding pathogenetic links between obesity and dyslipidemia and subsequent ASCVD is complex and incompletely understood. The mechanisms contributing to dyslipidemia in the obese include exaggerated synthesis of very low density lipoprotein (VLDL) by the liver, impairment of lipoprotein lipase (LPL) and lipolysis, LDL-receptor downregulation, reductions in HDL-C, and its related changes in neutral lipid transfer by enzymes such as CETP and phospholipid transfer protein (PTP) [34,48]. Finally, adipocyte dysfunction or ‘adiposopathy’ of mainly visceral adipose tissue (VAT) with adipocyte hypertrophy, adipose tissue inflammation, adipocytokine release, and excess FFA secretion are all major factors involved in the development of dyslipidemia and cardiometabolic disease [34].

Free fatty acid (FFA) turnover is the balance between FFA inflow from adipose tissue into the blood and FFA outflow into tissues whose source of energy is FFAs, such as the heart or skeletal muscle [49]. Obese individuals have elevated levels of circulating free fatty acids (FFAs) [49,50], which is thought to be due to (1) a surplus of metabolically active FFA-secreting adipocytes and (2) due to a vicious cycle of obesity-associated insulin resistance; insulin normally inhibits FFA mobilization from adipocytes, but excess of visceral adipose tissue is associated with insulin resistance [51]. Adipocytes thus release higher amounts of FFAs through excessive lipolysis, which further elevates plasma FFAs concentrations. In morbid obesity, due to excess caloric intake, adipose tissue becomes dysfunctional and with more supply of lipids than there is capacity, lipolysis increases, releasing free fatty acid systemically. These FFAs over time cause excess deposition of TGs at visceral adipose tissue but also in ectopic locations such as the liver and perivascular tissues. The deposition of FFAs in the liver leads to nonalcoholic fatty liver disease (hepatosteatosis, NAFLD), which itself contributes to increased insulin resistance and further enhancement of dyslipidemia [52]. VLDL hypersecretion occurs as the liver is meant as a glycogen-storage organ, not as a lipid- or TG-storing organ as excessive TGs can be seen as hepatotoxic. For this reason, excessive TGs are released into the circulation as VLDL, significantly contributing to raised triglyceride levels in obesity and especially in insulin-resistant patients of patients with fatty liver disease [22,52]. Apo-B-containing lipoproteins clearance is also reduced in NAFLD [53]. One of the drivers of NAFLD and ASCVD might be an altered gut microbiota composition [54].

LPL-mediated hydrolysis of triglycerides is the rate-limiting step for FFA transfer into tissues [55]. Studies have found that in obesity, there is impaired LPL activity and lipolysis, reducing the removal of postprandial FFAs and contributing to dyslipidemia through upregulated hepatic VLDL release which increases TG levels [23,56]. Finally, obesity has been associated with higher levels of CETP activity, enhancing the obesity-associated dyslipidemia with decreases in HDL-C and increases in the apo-B-containing lipoproteins [57,58].

## 3. Gut Microbiota Composition in Obesity and Dyslipidemia

Starting from the study in mice by Bäckhed et al. in 2004, which found that the introduction of gut microbiota into germ free mice increased total body fat by 57% despite diminished food intake [59], there has been substantial interest in the role of gut microbiota composition in the development of obesity. Later findings supporting the concept include the finding of obesity transmissibility through fecal matter transplantation, as was shown by Ridaura et al., where germ-free mice colonized with obese human fecal matter gave a significantly (*p* ≤ 0.001) higher fat mass and cohousing of lean and obese mice resulted in a lower change in adiposity, further suggesting transmissibility of traits such as obesity through the GM [60].

### 3.1. Gut Microbiota Composition and Dyslipidemia

The quest to identify a ‘healthy set’ of organisms amongst our gut microbiota has proven challenging, if not futile, at this moment and with the current knowledge. GM composition appears to change in time, vary amongst individuals despite a similar diet, and it depends on age, genetics, and environment [61,62,63]. Even some generally considered ‘pathogenic’ or ‘dysbiotic’ organisms are found to possibly be advantageous in other disease states [64]. The most commonly described change in GM ecology is the changes in the relative abundance of the bacterial phyla of *Bacteroidetes* as compared to *Firmicutes* in obese individuals and the increased *Firmicutes*-to-*Bacteroidetes* ratio as a predictor of obesity and cardiometabolic disease, observed in both murine models as well as in human studies [65,66,67]. However, further studies have shed a critical light on GM ecology as a contributing factor on itself in the development of cardiometabolic disease and obesity; in a study by Magne et al., the *Firmicutes*-to-*Bacteroidetes* ratio has been disputed as a predictor of obesity, and the study also underlined the complexity of associating this ratio with any existing health status [68]. As was written in another review by Tang et al. of related thematic, a ‘knowledge gap’ has developed due to research extensively attempting to portray the ‘healthy’ and ‘unhealthy’ GM ecology, whilst its produced metabolites, components, and accompanying functional changes have been overlooked to a certain extent [69]. Still, several studies have found changes in GM composition in dyslipidemia, indicating the possible role of GM in the regulation of lipid metabolism as is described in Table 1.

### 3.2. Coprostanol

One of the earliest findings, which possibly indicates a role of GM metabolites in cholesterol metabolism, was found by a study in 1934 that identified that cholesterol transformation into coprostanol by bacterial hydrogenation was a process performed by bacteria in the colon because it did not occur after sterilization of the intestinal contents [75]. Coprostanol is the reduced form of cholesterol which cannot be absorbed and is eliminated through the feces. Furthermore, individuals can be divided into low cholesterol-to-coprostanol converters and high cholesterol-to-coprostanol converters giving a bimodal distribution [76,77]. Few studies to date have explored the ability of GM to lower cholesterol levels through transformation of cholesterol into coprostanol by microbial enzymes. One recent study from 2020, by combining metagenomics and metabolomics data from several large study cohorts, identified microbial genes encoding a family of cholesterol dehydrogenase enzymes converting cholesterol into coprostanol, which they named the intestinal sterol metabolism A (*IsmA*) genes [78]. Furthermore, the study also found that individuals with gut microbiota producing these dehydrogenases not only had diminished levels of fecal cholesterol but also lower total cholesterol in the serum [78]. Another study found the rate of cholesterol to coprostanol conversion to be correlated with bile acids or SCFA secretion and identified 16 fecal metabolites in correlation with cholesterol conversion status [79]. The exact role of coprostanol formation in exogenous and endogenous cholesterol metabolism or the pathogenesis of dyslipidemia remains poorly understood to this day almost a century after its discovery.

## 4. Gut-Microbiota-Derived Metabolites in Dyslipidemia and Obesity

**Figure 2:**
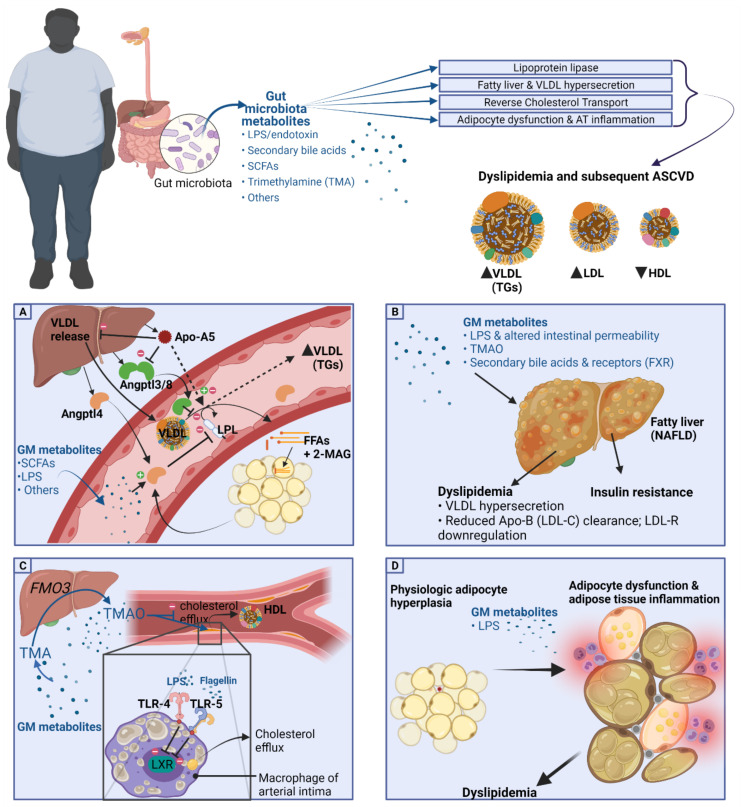
Gut microbiota and its metabolites in dyslipidemia. GM-produced metabolites have been associated with alterations of lipoprotein lipase function, fatty liver disease (NAFLD), inhibition of reverse cholesterol transport (RCT), and adipocyte dysfunction or adipose tissue inflammation. (**A**) Lipoprotein lipase (LPL) and GM metabolites: Studies have found that proteins which alter the function of LPL, including Angptl4, Angptl3/8, and Apo-A5, are acted upon by certain gut metabolites including SCFAs, LPS, and others, causing hypertriglyceridemia. (**B**) NAFLD and VLDL hypersecretion associations with gut metabolites. (**C**) Inhibition of reverse cholesterol transport and HDL-C by gut metabolites, primarily LPS, flagellin which upon binding to TLR-4 and TLR-5, respectively, cause inhibition of cholesterol efflux from intimal macrophages. Further metabolites include TMA and hepatically produced TMAO which possibly inhibit cholesterol efflux directly but rather contribute to ASCVD through ramping up the degree of cholesterol deposition in arterial intima macrophages, possibly through upregulation of scavenger receptor A1 and CD36 which collect oxidized LDL, and with further generation of foam cells, cause ASCVD. (**D**) Adipocyte dysfunction and adipose tissue inflammation in association with gut metabolites. SCFA, short-chain fatty acids; LPS, lipopolysaccharide; Angptl4 and 3, angiopoietin-like protein 4 and 3; Apo-A5, apolipoprotein A5; FFAs, free fatty acids; 2-MAG, 2-monoacylglycerol; VLDL, very low-density lipoprotein; LDL-C, low-density lipoprotein cholesterol; HDL, high-density lipoprotein; FXR, farnesoid X receptor; LXR, Liver X receptor α and β. TLR-4 and 5, Toll-like receptor 4 and 5.

There has been substantial evidence of a role of the gut microbiota and its metabolites in the pathogenesis of cardiometabolic disease and dyslipidemia. The aim of metabolomics is the identification of (GM-derived) metabolites in the plasma, bodily fluids, and within cells and correlates these with disease states. In the past decade, the primary method of choice to analyze metabolites in disease states has been high-frequency nuclear magnetic resonance (NMR) analysis and spectroscopy [80]. More recently, low-frequency NMR was also shown to be an easier-to-use but reliable method of identifying metabolite profiles in disease states such as T2DM [80]. Point-of-care applications of metabolomics is not possible as of now but might be so in the future, for example in assessing T2DM risk based on metabolite profiles [81]. A depiction of the role of GM metabolites in dyslipidemia and obesity can be seen in Figure 2.

### 4.1. Lipoprotein Lipase and GM Metabolites

As discussed earlier, LPL-mediated TG hydrolysis is the rate-limiting step for FFA transfer into adipose and other tissues, and impairment of LPL-mediated lipolysis is one of the possible pathogenetic mechanisms leading to dyslipidemia in the obese population [55,56]. In mice, there have been studies implicating a role of GM or its metabolites in regulating LPL function [59]. LPL function is further regulated by adipokines and other signaling molecules such as angiopoietin-like proteins (ANGPTLs) [82] and apolipoproteins including Apo-A5 [83], and there are findings that the gut microbiota and its metabolites influence these and in such a way regulate LPL-mediated lipolysis, causing shifts in triglyceride (TG) trafficking and subsequent dyslipidemia, as is summarized in Figure 2A.

#### 4.1.1. Angptl4 and Angptl3

Of the regulating factors of LPL function, the most relevant for the current discussion is angiopoietin-like protein 4 (Angptl4). Gut microbiota have been found to alter LPL function via modulation of a protein secreted by adipocytes and liver cells, termed Angptl4, or previously called ‘fasting-induced adipose factor (FIAF)’ [84]. Angptl4 appears to play a role in lipid metabolism via inhibition of LPL activity, thus decreasing the ability of adipocytes to take up TGs, causing hypertriglyceridemia [82,84]. Studies in mice have shown that recombinant Angptl4 injection or the induction of Angptl4 overexpression in transgenic mice increases TG levels in the plasma via inhibition of LPL activity, decreasing lipolysis of TG-rich lipoproteins such as VLDL [85,86,87,88]. The proposed function of Angptl4 is to provide fuel supply during fasting through inhibition of TG clearance and induction of lipolysis, thus increasing FFA levels in the plasma [89]. In the postprandial state, Angptl4 was shown to be inhibited by insulin, giving higher LPL activity, and thus, Angptl4 allows for the uptake of TGs from lipoproteins rich in TGs such as VLDL and chylomicrons and subsequent storage in adipocytes [89,90]. Joosen et al. showed that Angptl4 expression was lower in subcutaneous adipose tissue of study participants who were overeating, especially after the intake of the PPARγ ligand rosiglitazone, reiterating Angptl4′s role in postprandial metabolism [91]. In humans, the Angptl4 association with TG levels remains a topic of discussion; one study by Smart-Halajako et al. did not find an association between genetic variants of Angptl4 and lipid levels or CVD [92]. A later genome wide association study by Dewey et al., however, found that the loss of function of Angptl4 (missense E40k variant) was associated with reduced triglyceride levels as well as lower coronary artery disease risk [93].

Studies connecting GM or its metabolites to LPL activity through action on Angptl4 are limited, and up till now, primarily intestinal Angptl4 regulation of lipase activity has been found to be affected by gut metabolites; Angptl4 appears to inhibit not only LPL at the adipose tissue, but also pancreatic lipase in the intestinal lumen [94]. Gut microbiota through their metabolites including SCFAs are possibly altering Angptl4 action, as was identified by a study which concluded that in human intestinal cell lines the SCFA butyrate induces Angptl4 function independent of PPARγ [84]. One study found that colonization of germ-free 8–10 week old mice by microbiota from adult conventionally raised mice (‘conventionalization’) gave a 57% increase in fat tissue, a 122% (*p* < 0.001) augmentation in LPL activity in white adipose tissue, a 99% (*p* < 0.01) increase in heart tissue, and a drop in the LPL-inhibiting adipokine Angptl4 in the small intestines but not in adipocytes [59]. In vitro studies in cell lines have shown that SCFAs can increase Angptl4 synthesis [95], and mice colonization with the SCFA (including butyrate)-producing bacteria *Clostridium tyrobutyricum* was also able to increase Angptl4 in the mouse model mentioned [84]. In humans, few studies have investigated Angptl4 modulation by GM through SCFA or other metabolites. One study failed to illustrate associations between changes in the microbiota and Angptl4, did not find an influence of changes in diet on Angptl4 concentrations, and did not find a correlation between Angptl4 and plasma lipid levels [96]. However, this study investigated 18 participants, and larger studies evaluating the effect of GM or its metabolites including SCFAs on Angptl4 are therefore likely of added value.

Besides Angptl4, Angptl3 is another inhibitor of LPL, and loss-of-function mutations of Angptl3 are correlated with lower levels of TGs and cholesterol, and Angptl3 genetic deficiency is protective against ASCVD; thus, pharmacologic inhibition of Angptl3 is under current investigation as promising lipid-lowering therapy [97,98,99]. GM-derived LPS appears to alter Angptl3 function, as administration of LPS in mice caused a diminished Angptl3 expression [100]. Moreover, little is known about microbiota alteration of Angptl3.

#### 4.1.2. Apo-A5

Another regulator of LPL activity is Apolipoprotein A5 (Apo-A5), mainly derived from the liver and to a lesser degree from intestinal enterocytes. Apo-A5 is known to decrease plasma TG levels as certain variants of the *APOA5* gene have been associated with hypertriglyceridemia [101]. Although the main mechanism of Apo-A5 appears to be its ability to regulate LPL, other modes of action have been proposed such as reducing VLDL release by the liver, enhancing uptake of TG-containing particles, or by binding to the Angptl3/Angptl8 complex to cancel out its inhibition of LPL, causing an increase in LPL activity to lower plasma TG levels [83]. Cell cultures have found that SCFAs such as butyrate lead to higher Apo-A5 expression in vitro [102]. Another study found that the single-nucleotide polymorphism (SNP) of Apo-A5 minor allele, SNP rs651821, which is associated with hypertriglyceridemia, was also correlated with several microbial taxa including reductions in Actinobacteria such as *Bifidobacterium*. This implies influence and interaction between host genome and GM in metabolic syndrome and dyslipidemia, a relatively unexplored area [103].

### 4.2. Fatty Liver and VLDL Hypersecretion Association with GM Metabolites

Free fatty acids (FFAs) are esterified into triglycerides (TGs) for storage. The liver releases TGs into the circulation packed inside particles of VLDL, and together with enterocyte-derived chylomicrons, VLDL and chylomicrons are the main carriers of measured TG levels in the plasma. Nonalcoholic fatty liver disease (NAFLD) is strongly connected with obesity and is defined as a spectrum of liver disease including steatosis, steatohepatitis, fibrosis, and cirrhosis in order of severity occurring in the absence of alcohol use [104,105]. Hepatic steatosis occurs if FA inflow by hepatic FFA uptake and storage as TGs as well as de novo lipogenesis exceeds FA breakdown (oxidation) or outflow (VLDL export) [104]. NAFLD’s role in dyslipidemia is thought to be through VLDL hypersecretion and altered clearance of Apo-B-containing lipoproteins including LDL-C [53]. A complicating factor of studies assessing the role of NAFLD in cardiometabolic disease is the question whether TG accumulation in the liver (steatosis) as seen in NAFLD is the cause or effect of metabolic derangements such as dyslipidemia and insulin resistance, or even both. In NAFLD, there is often co-occurrence of substantial visceral adipose tissue (VAT) expansion and ectopic fat accumulation [106]. Furthermore, considering the role of insulin in TG delivery to the liver through regulation of several pathways including LPL-mediated lipolysis, insulin resistance and T2D are also big contributors to hepatosteatosis [107]. As mentioned, Apo-A5 is a regulator of VLDL secretion, and Apo-A5 itself is possibly regulated through GM metabolites. Several taxa have been found to be associated with NAFLD, and the reviews addressing the role of GM and metabolites in NAFLD as well as it interventions can be found elsewhere; potential pathways appear to be through microbial LPS, altered intestinal permeability, TMAO, and secondary bile acids and their receptors such as FXR (Figure 2B) [54,108,109].

### 4.3. HDL-C, Reverse Cholesterol Transport, and Cholesterol Transfer (CETP) Association with GM Metabolites

The reverse cholesterol transport (RCT) discussed earlier ensures cholesterol efflux from tissues including vessel walls to be returned to the liver. Liver X receptor (LXR) α and β besides a possible role in hepatic TG accumulation appear to regulate cholesterol efflux from macrophages onto HDL-C [110]. A 2003 study indicated a role of GM in RCT as they showed that for in vitro-cultured macrophages, toll-like receptors 4 (TLR-4; activated by gram-negative bacteria-derived lipopolysaccharide) and 5 (TLR-5; activated by viral RNA) both inhibit LXR signaling and thus interrupt cholesterol efflux from macrophages (Figure 2C) [111]. However, the importance of LXR in lipid metabolism has been undermined by the finding that inducing LXR pharmacologically was not effective in altering lipids and that LXR activation by agonists did not contribute to RCT [112].

Besides GM modulation of LXR signaling, the primary GM metabolite which was found to alter reverse cholesterol transport (RCT) was the oxidized form trimethylamine, TMAO. TMA is a GM-derived metabolite produced from L-carnitine, choline, or phosphatidylcholine from our diet and is converted to the oxidized form TMAO by the liver [113]. A study in mice by Koeth et al. found that a diet high in choline or L-carnitine, giving high TMAO levels, reduced reverse cholesterol transport (RCT) activity, reducing cholesterol efflux from the periphery, and reduced the expression of bile acid transporters, causing decreased cholesterol elimination [114]. The study also showed that eliminating TMA-producing bacteria by administration of broad-spectrum antibiotics gave lower TMAO levels and also reversed the decrease in RCT as was induced by the choline- or L-carnitine-rich diet [114]. Another study found that an increase in the activity of the enzyme converting TMA into TMAO, flavin monooxygenase isotype 3 (FMO3) was associated with changes in RCT as the knockdown of FMO3 in their murine model improved RCT and cholesterol balance [115]. Interestingly, in connection with LXR discussed above, the FMO3 knockdown also increased LXR-mediated cholesterol efflux from macrophages, ameliorating dyslipidemia in mice [115].

Studies investigating the effects of GM or metabolites on CETP function are few; the idea behind CETP in RCT is that higher function of CETP (as was determined by genome wide association studies) would lower HDL-C and thus worsen dyslipidemia. One study therefore investigated a possible connection between the TMAO and CETP in 394 coronary artery disease patients and 153 control subjects but found no association between TMAO and CETP polymorphisms associated with its function [116].

The first step of HDL synthesis is the formation of Apo-AI by hepatocytes or enterocytes, and an interesting recent finding is that Apo-AI synthesis is inducible through action on Toll-like receptor 5 (TLR-5), illustrated in a high-fat diet mouse model [117]. Besides LPS, flagellin is another pathogen-associated molecular patterns (PAMPs) and binds to Toll-like receptor 5 (TLR-5), and upon binding, flagellin causes release of proinflammatory cytokines [118]. The finding of hepatic TLR-5 and inducibility by a high-fat diet could possibly be a protective mechanism preventing metabolic derangements such as dyslipidemia after consumption of fatty meals, although this is only hypothetical for now.

### 4.4. Gut Microbiota and Bile Acids in Dyslipidemia

It is clear that bile acid formation is important in lipid metabolism, as the eventual removal of cholesterol occurs in the liver where cholesterol is converted into the primary bile acids chenodeoxycholic acid (CDCA) and cholic acid (CA) through hydroxylation reactions [119]. Next, primary bile acids are conjugated with glycine or taurine and excreted into the biliary tree to eventually reach the intestinal lumen where they can be acted upon by gut microbiota to form the secondary bile acids [119,120]. Although the majority of secreted primary bile acids are reabsorbed by terminal ileal enterocytes and (enterohepatic circulation), a proportion is not reabsorbed and reaches more distal parts where gut bacteria can dehydroxylate the primary bile acids, forming the secondary bile acids lithocholic acid (LCA), deoxycholic acid (DCA), and others [120]. Secondary bile acids are ligands for several bile acid receptors, including farnesoid X receptor (FXR) and G protein-coupled bile acid receptor-1 (GPBAR-1).

#### 4.4.1. FXR

FXR is expressed by several tissue cells including hepatocytes, adipocytes, intestinal cells, and pancreatic islet cells, and its most potent ligand is the primary bile acid CDCA acid, but it is also acted upon by secondary bile acids such as DCA. Since the early 2000s, FXR has already been shown to have important roles in lipid metabolism [121,122,123,124], and further, FXR knockout in mice gave hypertriglyceridemia and elevations in total cholesterol [125]. Hepatic FXR activation has been shown to decrease TGs systemically through lowering hepatocyte expression of sterol regulatory element-binding protein 1c (SREBP-1c), which is a transcription factor for de novo lipogenesis in the liver [121]. FXR activation also was found to ameliorate dyslipidemia by activating FFA oxidation via PPARα activation (also the gene-expression pathway for inducing HDL synthesis [122,126]), as well as by increasing apolipoprotein C-II activity [124], and decreasing hepatic VLDL production [123]. These beneficial effects of FXR on dyslipidemia sparked interest in the use of FXR agonists to treat not only triglyceride contents in the liver (NAFLD) but also dyslipidemia or hypertriglyceridemia; however, a randomized controlled trial assessing 72-week treatment with the FXR agonist obeticholic acid versus placebo in noncirrhotic NASH patients improved liver fibrosis, but total and LDL serum cholesterol was increased, while HDL-C decreased [127]. Novel trials assessing FXR modulation pharmacologically with beneficial effects on dyslipidemia are under way [128].

#### 4.4.2. GPBAR-1

GPBAR-1 or Takeda G protein-coupled receptor 5 (TGR5) is another bile acid receptor and is primarily activated by LCA and DCA, both secondary and GM-derived bile acids [129]. Bile acid signaling through GPBAR-1 appears to be more involved in bile acid pool modulation, biliary epithelial permeability, energy metabolism, glucose homeostasis, and inflammation, rather than in lipid metabolism directly [130]. The only identified connection between GPBAR-1 and lipid metabolism might be the finding that GPBAR-1-null (knockout) mice are found to be relatively protected from cholesterol gallstones and that patients with cholesterol gallstones exhibit higher GPBAR-1 mRNA expression levels in their gallbladder [131,132]. Although direct regulation of lipid metabolism seems unlikely to occur through GPBAR-1, it is important to be reminded that since bile acids are the emulsifiers of ingested triglycerides and fats and GPBAR-1 appears to regulate bile acid pool and secretion, GPBAR-1 is likely to play an indirect role in lipid metabolism, possibly through modulation of lipid uptake from the gut lumen.

### 4.5. Short Chain Fatty Acids and Dyslipidemia

Short-chain fatty acids (SCFAs) are produced in the gut lumen and are water soluble, hence being readily transported across membrane transporters in enterocytes to reach the portal blood [17]. The SCFAs present with the highest concentration in the human gut are acetate, propionate, and butyrate [133]. SCFAs are produced by commensal microbiota through fermentation of indigestible non-starch polysaccharides (dietary fibers) and can function as energy source for luminal microbes themselves, can be absorbed, and used for enterocyte energy, and finally, SCFAs can be present in low amounts in the circulation and supply energy to peripheral tissues being substrates for hepatic gluconeogenesis or adipocyte lipogenesis [134]. Importantly, however, SCFAs when present in the circulation act on several receptors throughout the body, such as G protein-coupled receptors, also termed free fatty acid receptors GPR41 (FFAR3) and GPR43 (FFAR2), olfactory receptors such as Olfr78, and others [135,136,137]. Higher amounts of SCFA production by the GM are increasingly seen as beneficial versus cardiometabolic disease.

As mentioned earlier, cholesterol-to-coprostanol conversion has been correlated with SCFAs [79], and SCFAs can alter LPL function through Angptl4 [84] or Apo-A5 [102]. Furthermore, stimulation of the SCFA receptor GPR43 in a mouse model and in vitro methods caused a decrease in adipocyte lipolysis, lowering free fatty acids in the plasma [138], and Hong et al. found that two SCFAs induce adipogenesis through the same receptor [139]. The inhibition of lipolysis by SCFAs was also confirmed by a study incubating adipocytes with SCFAs as they found that enhanced hormone-sensitive lipase (HSL) activity was responsible for decreased adipocyte lipolysis [140]. SCFAs supplementation has the ability to lower total cholesterol and triglycerides in several murine studies and cell models [141,142,143]. The cause of this beneficial effect is not completely understood, but according to Zhao et al., it might be due to SCFAs’ ability to increase new bile acid synthesis as SCFAs lead to higher bile acid losses and as a balancing mechanism bile acid synthesis increases, requiring more cholesterol from the circulation hence decreasing total cholesterol in the plasma [144].

### 4.6. TMAO and Lipid Metabolism

The tertiary amine trimethylamine (TMA) is the gut-microbiota-derived metabolite produced from L-carnitine, choline, or phosphatidylcholine from our diet and is converted to the oxidized form TMAO by the liver enzyme flavin monooxygenase isotype 3 (FMO3) [113]. Original findings were that TMAO worsened dyslipidemia by, for example, reduction of reverse cholesterol transport as mentioned before [114] and that TMAO feeding increased lipid levels in mice [145]. Other studies have revealed the exact opposite, as one study in rats fed a high-fat and high-carbohydrate diet and TMAO found that TMAO supplementation decreases cholesterol absorption in the intestine through modulation of transporter proteins and decreased hepatic cholesterol indicating positive roles of TMAO [146]. TMAO is now thought to not affect plasma levels of lipids directly but rather exert its proatherogenic effects by ramping up the degree of cholesterol deposition in peripheral tissues such as the arterial intima macrophages, possibly through upregulation of scavenger receptor A1 and CD36, which collect oxidized LDL, and with further generation of foam cells, cause ASCVD (Figure 2C) [113,147]. More recent findings also explain the increased CVD risk of TMAO (in part) by its ability to augment platelet hyperreactivity and increased atherothrombosis risk, rather than its role in lipid deposition and metabolism alone [148].

## 5. Adipocyte Dysfunction and Gut Metabolites

Adipose tissue is increasingly understood as an important endocrine and metabolically active organ, storing energy efficiently and releasing modulatory adipokines such as adiponectin. Healthy adipose tissue expansion is that of hyperplasia and adaptation; maintaining vascularization, increasing insulin sensitivity partly through adiponectin release, and allowing storage of triglycerides in a ‘healthy’ fashion, ready for release whenever energy is required [149]. However, in case of chronic overfeeding and obesity, adipose tissue does not only undergo physiologic hyperplasia, but it also hypertrophies [150]. Hypertrophic adipocytes do not store energy efficiently, but rather they are overloaded with triglycerides and excessively large cytoplasmic lipid droplets, and upon closer investigation, these enlarged adipocytes have inadequate vascularization, deteriorated mitochondrial oxygen use, hypoxia despite increased hypoxia-inducible factor 1-alpha (HIF1α), and as a result substantially increased reactive oxygen species (ROS) production, lipid peroxidation, and tissue fibrosis [149,150,151,152]. Hypertrophic adipose tissue does not allow for normal adipogenesis. With an increasing number of adipocyte progenitors over mature adipocytes, there is a decreased normal storage of TGs, inducing lipolysis and ectopic fat accumulation including in the liver [153,154].

Of the different types of adipose tissue frequently studied in humans, including subcutaneous and visceral adipose tissue (VAT), it is dysfunctional VAT which is believed to be the strongest driver of dyslipidemia, atherosclerosis, and cardiometabolic disease [150,155]. Intra-abdominal fat tissue (incl. VAT) exhibits higher lipolytic and inflammatory activity compared to subcutaneous fat tissue, which may explain that studies have consistently found a stronger association between intra-abdominal (visceral) fat and dyslipidemia and ASCVD than other fat tissue stores [155].

It appears that adipocyte hypertrophy, hypoxia, and a shift toward a proinflammatory adipose tissue drives the development of dyslipidemia and insulin resistance [150,156]. Adipocyte hypertrophy and dysfunction induce inflammatory changes including macrophage infiltration into fat tissue and macrophage subtype switch from M2 toward M1 macrophages, which are deemed proinflammatory [157]. Hypertrophied adipocytes secrete higher amounts of cytokines (adipokines) causing more inflammatory cell recruitment, including M1 macrophages [158]. The changes in adipocytokine secretion by mainly visceral adipocytes contribute to dyslipidemia, low-grade systemic inflammation, and ASCVD. Adipose tissue in obesity is characterized by higher levels of the proinflammatory cytokines, adipokines, and C-reactive protein (CRP), and lower levels of the anti-inflammatory and modulatory proteins such as adiponectin [159]. Figure 2D illustrates the mentioned changes and the associated gut-derived metabolites.

### 5.1. GM Metabolites and Adipose Tissue Dysfunction

In connection with impaired adipogenesis and increased preadipocytes over mature adipocytes seen in adipose tissue dysfunction, a study found that the GM-derived lipopolysaccharide (LPS) application on adipose tissue cells caused diminished adipogenesis as well as increasing premature senescence of adipocytes progenitors [160]. Other GM metabolites besides LPS have also been found to regulate adipocyte function, such as tryptophan-derived indole compounds produced by the GM, which was found to coordinate the expression of microRNA of the *miR-181* family, which are regulating factors for adipocyte function [161]. The indole compounds inhibited adipocyte differentiation and TG accumulation, mainly through the *miR-181* family of microRNA [161]. Finally, the endocannabinoid system (ECS) which is altered by GM, appears to regulate adipogenesis and energy balance [162,163]; however, little is known about the connection between the ECS and dyslipidemia.

Adipose function might also be related to gut microbiota through the TMA-FMO3-TMAO axis. The cross-sectional study on Finnish men (METSIM, *n* = 10,197) which performed adipose tissue biopsies on 1400 participants [164], together with other validation cohorts [165,166], formed the basis for a study by Schugar et al., which found a positive correlation between FMO3 expression in human adipose tissue and obesity [167]. Moreover, by performing further analysis in mice and humans, they revealed a role for brown or beige adipose tissue; they found that adipocyte FMO3 mRNA had inverse correlation with gene expression patterns of beige or brown adipose tissue in humans, and finally, via *FMO3* knockout in mice, they found higher amounts of beige adipose tissue and thus increased energy expenditure [167]. Brown and beige adipose tissue are increasingly described tissues involved in energy balance, lipid metabolism, and obesity in humans. It appears beige adipocytes are unique thermogenic cells distributed amongst white adipose tissue cells of mice and humans and have the capability to perform phenotype switch from white to brown adipose tissue in a process called ‘browning’ [168]. Brown and beige adipose tissue promote energy utilization by uncoupling cellular respiration from ATP synthesis and switch to thermogenesis (adaptive thermogenesis), while they increase fatty acid β-oxidation [169]. The importance of brown and beige adipose tissue in systemic metabolism was more recently highlighted by a publication that by using metabolomics proved that several metabolites are able to decrease adiposity, boosting energy expenditure, and improve glucose metabolism in mice [170]. Whether other gut metabolites than TMAO are actors in inducing browning of adipose tissue thus causing obesity or lipid metabolism disturbances remains to be seen.

### 5.2. GM Metabolites in Adipose Tissue Inflammation

Recent research has pointed toward a role of gut microbiota metabolites in chronic low-grade systemic inflammation [171], and it appears metabolite-induced inflammation does not only occur in the vessel wall as is seen with the metabolite TMAO [66] but also might be through inducing inflammation in our adipose tissue.

One of the earliest studies indicating that adipocytes themselves contribute to inflammation in obesity was provided by Hotamisligil et al. in 1993. As this group observed that white adipose tissue of obese mice with insulin resistance had elevated expression and synthesis of the inflammatory cytokine TNF-α, they also found that by neutralizing the elevations in TNF-α by administering anti-TNF antibodies, there was substantial improvement of insulin resistance [172]. Adipose tissue inflammation is seen as an important part of the path in which obesity leads to cardiometabolic disease including dyslipidemia, T2DM, and ASCVD. Obesity independently elevates inflammatory markers such as CRP [47]. According to several studies, in obesity and physical inactivity, dysfunctional adipocytes start secreting higher amounts of the pro-inflammatory cytokines IL-1 (α and β subtypes), TNF-α, and IL-6 [34,173,174]. Besides CRP, glycoprotein acetylation (GlycA) is another marker of systemic low-grade inflammation [175]. Mokkala et al. in a recent cross-sectional study in 335 overweight and obese pregnant women explored the value of GlycA in reflecting low-grade systemic inflammation status and gut microbiota diversity [176]. They concluded that, as compared to measured hsCRP (high-sensitivity CRP), GlycA is a better marker evaluating GM diversity and low-grade inflammation. Besides the increased pro-inflammatory markers, obesity is often associated with a decrease in adiponectin, an anti-inflammatory adipokine deemed beneficial against dyslipidemia and important in maintaining glucose and insulin homeostasis [34,177,178]. Another important adipokine includes the ‘original’ adipokine leptin, which exhibits many roles including regulating food intake [179]. In the current context, it is important to also understand leptin as a proinflammatory molecule, stimulating the release of IL-6 and TNF-α in adipose tissue macrophages [180]. Interestingly, these and other proinflammatory cytokines themselves induce leptin release from adipocytes [181], creating a vicious cycle of inflammation and leptin secretion, especially when adipocytes are numerous and dysfunctional such as in obesity. More and more adipokines are being identified as well as acute phase reactants; this further underlines the role of adipose tissue as a metabolically and immunologically active endocrine organ and its possible contribution to dyslipidemia in obese but also normal-weight individuals.

Lipopolysaccharide (LPS or endotoxin) produced by gram-negative bacteria as part of its outer wall has long been known to cause systemic inflammation, as the lipid A component of LPS leads to the development of septic shock and disseminated intravascular coagulation (DIC) in case of overwhelming gram-negative bacteremia [182]. LPS is however also present in low amounts in our circulation in non-shock states as LPS has been measured in substantial amounts in our plasma but especially after meals, which is termed postprandial endotoxemia [183]. LPS works by interaction with toll-like receptor 4 (TLR-4) and CD14 abundantly present on the surface of neutrophils and macrophages, which upon activation stimulate the release of the inflammatory cytokines, TNF-α, IL-1, and IL-6 [182,183]. High fat diets such as seen in obese individuals have been found to give higher amount of endotoxemia in some studies [184] but have also been disputed by others [185]. An early study in 2007 by Creely et al., which investigated the effects of LPS on obesity and T2DM, found that proinflammatory adipokines such as TNF-α and IL-6 were significantly (*p* < 0.001) increased in cultured human subcutaneous adipose cells treated with LPS, and they underlined that LPS works via activating an innate immune response in adipose tissue [186]. Miller at al. in a study of 192 participants observed positive associations between endotoxemia levels and total cholesterol, serum TGs, and insulin, while they found a negative association between endotoxemia and HDL-C [187]. LPS has already before been found in the crossroads with lipid metabolism before; in states of sepsis, HDL seems to bind and neutralize LPS and furthermore, systemic inflammatory response disrupts lipid metabolism by reducing HDL levels and increasing cholesterol and TG levels, termed the ‘lipemia of sepsis’ [188]. Furthermore, LPS activity, as measured by the LPS-to-HDL ratio, was found to be strongly correlated with serum TG concentration as well as with chronic inflammation in a study by Lassenius et al. analyzing data from several large cohorts [189]. Whether LPS present in low amounts contributes to dyslipidemia in other ways than a drop in HDL secondary to HDL-mediated neutralization is undetermined; however, chronic low-grade inflammation seems to be a clear component of endotoxemia.

Finally, a more novel approach is the crude identification of bacterial DNA within adipose tissue itself. In a recent study by Massier et al., ensuring strict control for contamination, bacterial DNA was identified within several adipose tissue types including the phyla of *Firmicutes* and *Proteobacteria* [190]. They also found that the quantity of bacteria as well as its distribution within adipose tissue was correlated with the degree of adipose tissue inflammation [190]. Although the exact metabolites the GM produced within the adipose tissue were not investigated in this study, it highlights the possible role of GM in adipose tissue inflammation as seen in obesity, T2DM, and dyslipidemia.

## 6. Gut Microbiota Metabolites in Dietary, Weight Loss, and Pharmacologic Interventions

### 6.1. Dietary Interventions and Gut Microbiota

Covering the effects on dietary interventions on the GM and its metabolite production is beyond the scope of this review; however, the Mediterranean diet (MD) has received special attention in this regard. MD is a heart-healthy diet, beneficial against dyslipidemia (LDL-C reduction mainly) and decreasing CVD risk [191,192]. MD is a diet rich in fruit and vegetables, unsaturated fatty acids, wholegrain products, and overall contains high amounts of dietary fibers. Fiber-rich diets such as MD, vegetarian, or plant-based diets have been associated with substantial changes in the gut microbiota such as higher bacterial richness as compared to the Western diet which is rich in sugars, saturated fats, and contains fairly little dietary fiber [193]. In connection with dyslipidemia, one study randomizing 82 overweight and obese individuals for MD versus their regular Western diet while maintaining energy intake found that MD reduced total and LDL-cholesterol levels and that the reduction was correlated with fecal bile acid concentrations, increased bacterial gene richness, and certain shifts in GM composition [194]. The beneficial effects of dietary fiber are possibly through higher SCFAs production, as SCFAs are synthesized through fermentation of indigestible fibers by the commensal GM [134]. As the MD has substantial larger amounts of fibers, studies have found higher amounts of SCFA production with this diet, and furthermore the diet has shown to decrease *Firmicutes* while increasing *Bacteroidetes* phyla, which is relevant because *Bacteroidetes* is associated with increased SCFAs production [195]. Whether GM composition is merely a biomarker of the diet consumed is becoming less likely, as the diet rather appears to play an active role in altering GM composition and mediate its cardiometabolic-associated diseases.

### 6.2. Weight Loss Surgery and Gut Microbiota

As has been reviewed elsewhere [109,196,197], there is a relation between the gut microbiota or its produced metabolites and bariatric surgery such as laparoscopic Roux-en-Y gastric bypass (RYGB) or sleeve gastrectomy. Besides weight loss, bariatric surgery improves diabetes, hypertension, and NAFLD; furthermore, bariatric surgery, particularly RYGB, has been shown to greatly improve dyslipidemia in patients with obesity; and commonly observed lipid profile changes after bariatric surgery include reductions in total cholesterol and LDL-C, increments in HDL-C, and lowering of TG levels [198,199,200]. Whether the path by which bariatric surgery conveys beneficial changes in GM and metabolites also improves lipid metabolism is not known. One prospective study by Palmisano et al. found RYGB not only resolved hypercholesterolemia in 70.6% of patients, but also that after RYGB, there was a correlation between microbial composition and the achieved cholesterol improvement on unweighted and weighted distance matrices using BIOENV dissimilarity method [201]. Another study evaluating the role of GM in the success rate for several health parameters found that lipid metabolism was the parameter most associated with gut microbiota signatures after bariatric surgery and hypothesized that these benefits in lipid metabolism occurred due to changes in the bile acid milieu [202]. Chakaroun et al. investigated 64 individuals with and without T2DM undergoing bariatric surgery and identified a bacterial blood signature in correlation with features of the metabolic syndrome, formulating a metabolic syndrome classification index based on bacterial phylum and genus level abundances. The study also found that the weight loss and improvement of metabolic syndrome after bariatric surgery was associated with bacterial composition, quantity, and their identified taxonomic signatures [203]. The three mentioned studies investigated only 25, 24, and 64 individuals, respectively [201,202,203]; thus, larger studies are needed to evaluate whether benefits of bariatric surgery on dyslipidemia are, at least in part, through changes in gut microbiota or its metabolite production.

### 6.3. Lipid Lowering Agents and GM

Statins are the most widely used agents to lower LDL-C levels and work by blocking HMG-CoA reductase; however, substantial differences in treatment efficacies have been observed [6,204,205]. In an attempt to identify metabolite signatures, Kaddurah-Daouk et al., through targeted GC-MS metabolomics, characterized three secondary GM-derived bile acids able to anticipate the degree of the LDL-C-lowering effect of simvastatin therapy and correlated levels of secondary bile acids with simvastatin concentration [206]. The same study also revealed a correlation between coprostanol levels prior to treatment with simvastatin and the degree of LDL-C lowering in response to the statin [206]. Statins also appear to alter GM composition itself and potentially mediate some of its lipid-lowering effects through modulation of GM ecology. Vieira-Silva et al. found that the prevalence of *Bacteroidetes* 2 (*Bact2*) enterotype is lower in individuals taking statins; this *Bact2* enterotype with increased *Bacteroides* and decreased *Faecalibacterium* has been correlated with systemic inflammation, loose stools, and obesity [207,208]. Likewise, another study found increases in anti-inflammatory GM ecology after atorvastatin use in humans [209]. The effects of statins on GM and GM metabolites was further emphasized by a mouse study, which revealed that statin use modified GM diversity and profile, decreased production of the SCFA butyrate, changed the bile acid pool as well as energy metabolism, and that these effects might be through the pregnane X receptor (PXR) [210].

Fibrates have the substantial ability to lower triglyceride levels and, to a lesser degree, can reduce LDL-C while raising HDL-C [6]. Fibrates are agonists at peroxisome proliferator-activated receptor alpha (PPARα), a transcription factor, and fibrates thus upregulate ApoA-AI (HDL component), increasing LPL activity and fatty acid oxidation, and reduce VLDL production by the liver [211]. Although studies assessing the effect of fibrates on GM or its metabolites were not found, the receptor upon which fibrates work, PPARα, has been of interest in GM or GM metabolite studies. For example, high-dose SCFA acetic acid administration to mice showed increased PPARα gene expression [212], and GM modulation with the nondigestible fiber α-Cyclodextrin-modified GM but also increased PPARα gene expression [213]. Personalizing the choice of lipid lowering agents based on gut microbiota composition and predicting treatment efficacy is not possible as of yet, and it is not clear whether the beneficial effects of these drugs are also acting through GM metabolite modulation.

### 6.4. Supplementation and GM

Probiotics are a disputable means of altering the gut microbiota composition in terms of dyslipidemia treatment. One study published this year found that *Bacillus* sp. *DU-106* probiotic supplementation increased bacterial richness and decreased cholesterol in hypercholesterolemic rats [214]. In humans, a randomized control trial in 128 subjects with elevated TG levels without diabetes found that supplementation with a probiotic containing *L. curvatus HY7601* and *L. plantarum KY1032* for 12 weeks decreased triglyceride levels while increasing LDL-C levels and increasing Apo-A5 particle size [215].

Benefits of polyunsaturated omega-3 fatty acids (hereafter ‘omega-3s’) supplementation on the reduction of CVD risk (including coronary artery disease or cardiovascular events) appear to be modest according to the most recent and largest-to-date systematic assessment [216]. However, the ability of omega-3s such as eicosapentaenoic acid (EPA) and docosahexaenoic acid (DHA) to reduce triglyceride levels is a more consistent finding in studies throughout [217,218,219]. The mechanisms by which omega-3s lower TGs are incompletely understood, possibly acting on VLDL levels by enhancement of apoB-100 turnover, causing a reduction of ApoC-III levels and subsequent induction of lipoprotein lipase, or by the action of omega-3s on peroxisome proliferator-activated receptors (PPARs) [217,220,221]. The effects of omega-3s on the gut microbiota or the formation of GM metabolites need further research. A randomized trial of small size (22 healthy adults) investigated omega-3-containing capsules versus a liquid form and its effects on GM composition and found higher butyrate-producing microbes following omega-3 supplementation in a reversible manner [222]. A larger study was performed in 876 female twins in the United Kingdom, which found GM alpha diversity to be correlated with omega-3s in serum; furthermore, the study found an even stronger correlation between DHA and the operational taxonomic units in the GM including the *Lachnospiraceae* family [223]. A recent study, which randomized patients between a 6 week supplementation of omega-3s or the prebiotic fiber of inulin, found omega-3s to cause increases in the species of *Coprococcus* and *Bacteroides*, a decrease in hepatosteatosis-related *Collinsella spp.*, and significant increases in branched-chain fatty acids isobutyrate, isovalerate and near-significant higher levels of the SCFA butyrate; however, no significant association with alpha diversity was found [224].

## 7. Conclusions

We aimed to provide a thorough review on available literature regarding gut microbiota composition and its role on regulation of classical risk factors of cardiovascular disease including dyslipidemia, fatty liver disease, and adipose tissue dysfunction and inflammation. Moreover, we describe the emerging role of new gut-microbiota-derived metabolites including TMAO and SCFA. However, it is still not well known whether these metabolites are disease modifiers or truly causal drivers of dyslipidemia or cardiometabolic disease. Future research will have to focus on human intervention trials and large prospective cohorts aimed at altering these gut-microbiota-derived metabolites in order to reduce cardiometabolic risk.

## Figures and Tables

**Table 1:** Gut microbiota in relation to dyslipidemia.

Reference	Study Description	Subjects	Methods	Main Findings
Fu et al., 2015 [70]	Large cohort study	893 participants of the LifeLines-DEEP cohort	Lipid panels of total cholesterol, HDL-C, TGs, LDL-C. Sample genotyping. Fecal sample gut microbiota profiling with 16s rRNA gene sequencing. Operational taxonomic unit (OTU) picking and OTU richness. Microbial Shannon diversity index calculation. Cross validation analysis.	In total, 34 bacterial taxa correlated with plasma lipid levels and BMI. Specifically, family *Clostridiaceae/Lachnospiracease* was correlated with LDL. *Coprococcus* (*Firmicutes*) and *Collinsella* had strong correlation with TGs. Cross validation analysis revealed that GM explained ≤25.9% of HDL-C variance and that microbiota explained 6% of the variance in TGs.
Rebolledo et al., 2017 [71]	Case control study	30 hypercholesterolemic subjects, 27 controls with normal cholesterol levels	Anthropometric data. Serum fasting glucose and lipid profile. Stool sample analysis of GM by gel electrophoresis with denaturing gradient (DGGE) technique and nonmetric multidimensional scaling (NMDS). Shannon–Weaver, Simpson, and Richness microbial community diversity indices.	DGGE banding profiles differed between case and control, confirmed by 2 separate groups forming on NMDS scaling analysis. Significant (*p* < 0.05) decrease of all three bacterial DNA indices of microbial diversity.
Vojinovic et al., 2019 [72]	Prospective cohort study of two large cohorts.	2309 individuals from the Rotterdam and the LifeLines-DEEP cohort.	Fasting plasma metabolite profiling with ^1^H-nuclear magnetic resonance (NMR). Fecal sample gut microbiota profiling with 16s rRNA gene sequencing.	Significant (*p* < 0.05) associations between 18 families and genera of bacteria with VLDL of different sizes, 22 with HDL, 13 with HDL and VLDL, and 15 with serum triglycerides
Le Roy et al., 2019 [73]	Mouse study	Hypercholesterolemic female Apoe^−/−^ and LDLr^−/−^ mice	Depletion of all microbes in hypercholesterolemic mice with a combination of 4 antibiotics. Human feces intestinal microbiota transplantation. Further on mice: Plasma lipid and lipoprotein profile analysis. Bile acid synthesis measurement with labeled (^14^C) cholesterol dissolved in olive oil. Liver, ileum, and jejunum gene expression with qPCR. Sterol quantification in liver and bile. 16S rRNA gene sequencing of fecal gut microbiota.	Depletion of microbiota in mice raises cholesterol, mainly VLDL and LDL. Depletion also enhances liver uptake of cholesterol. Depletion increases cholesterol de novo synthesis by liver. Cholesterol level is transmissible in mice by microbiota transplantation from humans with altered cholesterol levels to microbiota depleted Apoe^−/−^ mice.
Yun et al., 2020 [74]	Cross sectional study	1141 subjects from the Kangbuk Samsung Health Study in South Korea, divided into dyslipidemic (G0) and normal lipid (G1) groups based on total cholesterol, LDL-C, TG, HDL-C, ApoB and ApoA1 levels.	Anthropometric data. Blood sample after 10 h fasting of total cholesterol, triglycerides, LDL-C, HDL-C, and apoA1 (HDL particle component) and ApoB (LDL-C and other particle component) determination. Gut microbiota 16s rRNA gene sequencing of fecal sample.	The group with high TGs had lower alpha diversity indices (Shannon’s index and Faith’s phylogenetic diversity, both *p* < 0.001, Pielou’s evenness *p* < 0.030). Abnormally low ApoA1 group had higher alpha diversity. No association with alpha diversity for other lipid parameters. 12 taxa associated with TGs: The high TGs group had high amount of the genus *Fusobacterium* and low levels of *Oscillospira,* which produces butyrate (a SCFA) 10 and 6 taxa were associated with ApoA1 and ApoB levels, respectively.
